# Advancing mental health parity to ensure children’s access to care

**DOI:** 10.1038/s44184-024-00070-1

**Published:** 2024-06-13

**Authors:** Nathaniel Z. Counts, Ashwin Vasan

**Affiliations:** 1 New York City Department of Health and Mental Hygiene, Long Island City, NY USA; 2 Present Address: The Kennedy Forum, Brigantine, NJ USA

**Keywords:** Health policy, Health services, Psychiatric disorders

## Abstract

Mental health and substance use parity provides a rhetorical device and policy strategy for achieving more equitable financing of mental health and substance use services, which the U.S. has pursued as a lead policy approach for improving access to mental healthcare. Parity implementation in the U.S. has improved access to care for children, but implementation challenges remain, leading to persistent treatment gaps and disparities, workforce shortages, and variable care quality. In the U.S., a recent policy change required health insurers to make available all of the data on their coverage and reimbursement practices for all health conditions. This new data enables a more detailed conceptualization of what parity means in children’s mental health and how it should be implemented and overseen. Researchers, clinicians, and advocates across the globe can use this data to build the case and the policy approach for parity, supporting more equitable financing of children’s mental health and substance use care and promoting families’ access to evidence-based care.

In 2022, 19.5% of youth in the United States ages 12–17 reported having a past-year major depressive episode, but only 47.8% of those youth in need received treatment^1^. Youth also face substantial disparities in access to care, as White non-Hispanic youth were more than 50% likelier to receive treatment than their Black counterparts. These figures include all youth that received any amount of treatment—it does not indicate anything about the sufficiency of the treatment or its effectiveness relative to their needs. Globally, these disparities become even more stark, with youth facing even larger treatment gaps in most other countries and experiencing deep inequities in access within countries^2^.

The high level of need and gaps in treatment have not gone unnoticed, and U.S. policymakers have instituted reforms to expand access to care. One of the most central is the legal and regulatory concept of mental health and substance use parity. Parity in the U.S. is most critically enshrined in the Mental Health Parity and Addiction Equity Act (MHPAEA) of 2008. MPHAEA required that “the treatment limitations applicable to such mental health or substance use disorder benefits are no more restrictive than the predominant treatment limitations applied to substantially all medical and surgical benefits covered by the plan (or coverage) and there are no separate treatment limitations that are applicable only with respect to mental health or substance use disorder benefits”^3^. This means that, if an insurer does not impose limitations on coverage for conditions like diabetes and cancer (e.g., number of visits, criteria for whether a service is medically necessary, or caps on total costs), they cannot do so for mental health and substance use. While there are various exceptions to this, such as the ability to exclude all coverage for certain mental health conditions, this basic framework governs parity implementation and enforcement^4^. Today, as the result of subsequent laws expanding and building on parity, the requirements apply to many of the health insurers across the U.S., including managed care for Medicaid (the U.S.’s safety net health insurance system), employer-based insurance, and qualified insurance plans for individuals, and supports coverage for all beneficiaries and the providers that they see ref. ^5^. Because parity governs health insurance benefits, it has no impact on mental health and substance use care that families pay for entirely out-of-pocket.

In interpreting the parity statutes, the federal implementing agencies (i.e., the Department of Health and Human Services, the Department of Labor, and the Internal Revenue Service) have avoided taking a narrow view on what it means for a health plan to discriminatorily limit treatment. As part of what might limit the scope of a consumer’s access to treatment, federal agencies expansively include health plan policies and practices around the particular services covered, reimbursement rates for services, and even the amount of administrative burden put on clinicians^6^. This encompasses the written policies as well as the way they are implemented. Even if a plan has similar administrative requirements on paper for oncologists and psychiatrists, if the way the insurer implements the requirements is far more burdensome for psychiatrists, this would violate parity. The final parity regulation also notes that “commenters suggested that specific mental health or substance use disorder benefits be cross-walked or paired with specific medical/surgical benefits (e.g., physical rehabilitation with substance use disorder rehabilitation) for purposes of the parity analysis,” but ultimately rejected this in favor of a more holistic approach that acknowledges potential fundamental differences in the needs around treatment between mental health and other health conditions^7^. This means that restrictions around which providers may bill, what is required to bill, what services are covered, and how much these services are reimbursed must all fairly acknowledge the differences between mental health and other types of care.

Since 2008, parity has become the dominant paradigm for expanding mental health and substance use financing in the U.S., enjoying support from policymakers across the political spectrum. It has supported coverage and reimbursement reforms that can otherwise be difficult to implement, given that the U.S. relies on a patchwork of public and private health insurers to cover healthcare costs^8^. As a result of the parity laws, children and adolescents in the U.S. with access to covered health insurance plans enjoy the right to equitable coverage of mental health and substance use care. Although parity does not require coverage of a certain minimum set of services, it does require that mental health and substance use care not be unfairly limited, and gives families a set of tools to redress wrongful discrimination in coverage. With the parity law in effect, families can appeal denials with a transparent process for review, file complaints with regulators, and even sue.

Mental health and substance use parity may also offer a conceptual frame for advancing equitable children’s mental health financing across national contexts^9^. In countries with more robust healthcare financing systems, parity requirements can be a way to ensure fairness in the development and administration of coverage policies. In countries that are earlier in the process of developing comprehensive healthcare financing, parity could serve more as a rhetorical device and North Star to promote the fair inclusion of mental health and substance use care. In later stages, parity could translate into actual policy mandates that govern coverage and access, as robust systems of financing develop.

## Implementation of parity in children’s mental health

The parity requirements in the U.S. have achieved measurable gains in utilization of mental health and substance use treatment services^7^. For example, one analysis found that the federal parity law was associated with a 54% increase in spending on mental health and substance use services in July 2012 in a sample of children and adolescents^10^. Notably, the policies have achieved these gains despite only partial implementation. The complexity of health insurance in the U.S. means that oversight and enforcement will remain an ongoing process in the years to come.

The challenges with implementation are exemplified in oversight reports from federal agencies. After insurers have increasingly remedied many discriminatory quantitative treatment limitations, such as annual caps on the number of therapy sessions, agencies have focused oversight efforts on non-quantitative limitations, such as how an insurer decides on reimbursement rates or whether treatments are covered. In one oversight report, the agencies noted that “none of the comparative analyses submitted by plans or issuers during the Reporting Period were initially sufficient to satisfy provisions of MHPAEA,” which are needed to show that the insurers’ policies and practices do not unfairly limit treatment^11^. Only through “follow-up requests, continued conversations, insufficiency letters, and the possibility of being named as non-compliant in this report eventually incentivized a significant proportion of plans, plan sponsors, and issuers to correct MHPAEA violations.”

The implementation challenges may partially explain the gaps in access to mental health and substance use care that children experience in the U.S. Discrimination in which services are covered and at what rates can undermine the implementation of comprehensive team-based care models that advance equitable access, disincentivize providers from taking insurance, and even discourage people from entering the behavioral health workforce at all, potentially contributing to current shortages. While other factors also play a role in the access challenges that children face, comprehensive implementation of parity can ensure fairer financing that can defray some of the most pressing issues.

As part of the ongoing implementation to address these challenges, in 2023, the Biden Administration, through the lead federal agencies, proposed new regulation and released a request for information on what information is relevant for understanding parity in reimbursement and coverage, especially as it relates to network adequacy and the provider shortage facing the U.S^12–14^. The agencies’ initial proposal to assessing reimbursement and coverage parity included a few approaches. First, they could compare how plans reimburse for some key services, such as office visits for an established patient (CPT codes 99213 and 99214) or psychotherapy (CPT codes 90834 and 90837) for mental health and substance use providers as opposed to medical and surgical providers. They also proposed comparing how much plans reimburse for different services to a benchmark (e.g., payment rates from Medicare, a federal insurance program for older adults and disabled individuals directly administered by the U.S. government), to see if there are systematic disparities. Parity can apply to reimbursement and coverage in a number of ways, but this indicates how the lead agencies are currently thinking about oversight and enforcement. The agencies will likely decide on initial oversight strategies and begin implementation in 2024.

The timing of this inquiry is fortuitous because it builds on a related policy change. On January 1, 2022, a federal regulation went into effect requiring many health insurers to make available all of their negotiated reimbursement rates for every service they cover for every provider they contract with under every plan that the insurer offers, as machine-readable files^15^. This represents a profound change in transparency and data access. Before this, providers may know their own negotiated rates, but would have no way of knowing how much others were reimbursed. Now, all of this information is available to the public.

The data access is not without its challenges though. CMS provides technical guidance that is meant to make the data more accessible by standardizing it^16^. Even so, every service reimbursement rate for every healthcare provider for every health insurance product offered is an incredible amount of information that is difficult to make sense of. One major health insurer even warns, “Files are in a JSON format and may contain millions of lines of data and be up to 1 TB in size. Please consider your system’s capacity and memory when downloading these files”^17^. Programmers skilled in working with the relevant database formats are necessary to even begin to consider how to compare reimbursement rates. This must be paired with subject matter expertise to know what rates are relevant. Despite the challenges, the data provides a new opportunity to advance more equitable reimbursement and coverage for children’s mental health and substance use care.

## The particular opportunity in children’s mental healthcare

This set of policy changes opens important opportunities for advancing access to children’s mental health and substance use care. In the U.S., children’s mental health and substance use care is financed through narrowly defined units of service, such as psychotherapy or medication management for a set number of minutes. Increasingly, evidence-based care involves interdisciplinary care teams working together in flexible arrangements that meet the particular needs of a child, such as the collaborative care model, the primary care behavioral health model, and coordinated specialty care models^18,19^. These flexible models also support racial, ethnic, linguistic, and socioeconomic equity by creating more space for addressing needs specific to a community or cultural context^20^. Many innovative and promising practices are also built on the platform of these models, such as new technologies, behavioral interventions, or referral pathways implemented in the context of team-based care.

Team-based care is not unique to children’s mental health and substance use, but a core question arises as to whether all of the components of evidence-based children’s mental health care are covered and fairly reimbursed when compared to medical and surgical coverage. It is likely that the gaps in mental health and substance use treatment—and the likely but not well-documented gaps in quality—are likely at least in part a result of inequitable coverage and reimbursement that has been difficult to quantify until now^21^. For example, there is mounting evidence that clinicians find the collaborative care model difficult to implement and sustain under existing coverage and reimbursement policies without supplemental grant funding or without the resources and infrastructure of a large academic medical center, but data was often limited^22^.

In part because of the lack of data, many of the parity enforcement or litigation actions related to children’s mental health involve access to higher levels of care, such as residential treatment^23^. Without insight into coverage and reimbursement, enforcement and litigation has often not been able to address the fundamental financing of the children’s mental healthcare system, with attention to evidence-based practice in areas such as team-based care. The new data opens up a variety of new avenues for parity inquiry to begin to rectify this.

One way to use this new data is to focus on the services covered. Does an insurer’s policies cover aspects of care integration, coordination, or management in physical health but not mental health and substance use? Are rehabilitative or other supportive services offered for certain physical health diagnoses but not mental health and substance use diagnoses, even though they are equivalently evidence-based and medically necessary? Are there medically necessary services in children’s mental health and substance use that may not have a clear analog in physical health, but should be covered under parity? Again, cross-walking is not necessary—parity acknowledges the potentially unique aspects of mental health and substance use care in analyzing potential discrimination.

Another set of analyses could look at reimbursement rates for services. The federal agencies proposed several approaches on which researchers could build, considering the particular dynamics of child and adolescent mental health and substance use care. Approaches could look at the extent to which covered services and proposed reimbursement rates cover the cost of evidence-based models of children’s mental health and substance use care, such as coordinated specialty care, when compared to models in physical health care. Another could examine how the services covered and their reimbursement rates add up over time during the course of a normal workflow to determine whether the covered services fail to account for key activities needed for effective care that leads to discrimination in aggregate financing.

These strategies offer just a few possible examples of the many directions in which data analysis could advance more equitable coverage of children’s mental health and substance use care. Each inquiry also represents a potential violation of the parity law, as lack of coverage or lower reimbursement rates may represent treatment limitations on mental health and substance use benefits that are more restrictive than medical and surgical care. To the extent that these analyses lead to enhanced oversight that addresses under-reimbursement, this may translate into higher salaries in the behavioral health workforce and help build the workforce pipeline. If oversight addresses coverage gaps driving disparities in access, this may also help to build toward more equitable provision of care. Ultimately, with access to new data, stakeholders are better equipped to advance parity arguments that promote more systemic equity in financing that can address challenges facing children and families in accessing in mental health and substance use care.

## Next steps for advancing equitable coverage and reimbursement

New data offers new opportunities for researchers, clinicians, and advocates to advance the concept of parity in the U.S. and beyond, supporting access to evidence-based mental health and substance use care for children. Although the data is specific to the U.S., the analysis may still help with global questions about equitable financing in mental health and substance use care. This data offers the most detailed insight into the financing of any country that allows market forces to determine healthcare coverage and reimbursement (i.e., with coverage and reimbursement not set by public agencies). The data can support considerations, approaches, and arguments for equitable financing across countries by showing how analyses might be conducted or demonstrate why greater transparency in coverage and reimbursement is so important. The analysis could expand beyond the initial inquiries of the federal agencies to more comprehensively answer the fundamental question: What does equitable coverage and reimbursement for mental health and substance use care for children look like? As noted, for many countries, parity can serve as a goal and an important rhetorical device at the beginning, but eventually, all healthcare financing systems will need to determine the specifics of how to implement and oversee equitable coverage.

Researchers, clinicians, and advocates globally all have roles in using the new data to advance this aim. Researchers can illustrate how parity can be operationalized to ensure fair access to evidence-based care in the U.S. and abroad, and offer findings about how current coverage and reimbursement in the U.S. does or does not comply with parity requirements, potentially informing insurance design and oversight efforts. Clinicians in the U.S. can use this data to inform future contract negotiations with insurers or even parity complaints or other enforcement initiatives, enabling them to receive fair and sustainable financing for effective care. Advocates in the U.S. can use this data to urge greater oversight and enforcement across health insurers, illustrating gaps in current oversight while recommending new approaches. Part of the advocacy will also need to focus on data transparency and accessibility. As noted, the data now available in the U.S. is extremely difficult to interpret, and especially unusable for consumers. Standards for reporting information about healthcare financing, coverage, reimbursement, and costs would empower the movement toward parity and improve consumer and provider engagement in advancing equitable healthcare systems.

Internationally, this data can be used to illustrate how healthcare financing in any context can be more equitably allocated for children’s mental health, and to advance the call generally for parity as both a rhetorical device and a regulatory framework for achieving greater investment in children’s mental health. While parity has been a pillar of advocacy among organizations representing individuals with lived experience, their families, and their providers in the U.S., there is more work to be done advancing parity in international advocacy. Researchers, clinicians, and advocates can act as powerful partners in advancing parity globally.
